# Novel SNP in the coding region of the FTO gene is associated with marbling score in Hanwoo (Korean cattle)

**DOI:** 10.1186/2055-0391-56-27

**Published:** 2014-12-03

**Authors:** Eui-Ryong Chung

**Affiliations:** Division of Animal Science and Resources, College of Life Science and Natural Resources, Sangji University, 660 Usandong, Wonju, Gangwondo, 220-702 South Korea

**Keywords:** FTO, Marbling, SNP, Meat quality, Hanwoo

## Abstract

The *fat mass and obesity associated* (FTO) gene plays an important role in the regulation of energy homeostasis, fat deposition and obesity. For this reason, the FTO gene is a physiological and functional candidate gene for carcass and meat quality traits in beef cattle. The objectives of this study were to identify SNPs in the exonic regions of FTO gene and to evaluate the association of these SNPs with carcass traits in Hanwoo (Korean cattle). In this study, we newly identified two exonic SNPs in Hanwoo population. The g.125550A > T SNP was located in exon 3 and the g.175675C > T SNP was located in exon 6. Genotyping of the two SNP markers was carried out using PCR-RFLP analysis in Hanwoo steers to evaluate their association with carcass traits. As a result, g.125550A > T SNP genotype was significantly associated with effects on marbling score. Animals with the AA and TT homozygous genotypes had a significantly higher marbling score (*p* < 0.001) than those with AT heterozygous genotype, and this was significant after *Bonferroni* correction of the significance threshold (*p* = 0.003). Dominance effect was also observed for the marbling score (P < 0.05) with higher marbling score of homozygous animals. However, no significant associations with meat quality traits were observed for the g.175675C > T SNP. Our results suggest that the exonic SNP g.125550A > T in the FTO gene may be used as a DNA marker for the selection of Hanwoo with higher marbling.

## Background

Marbling is the most economically important trait in beef cattle industry of Korea. Generally, marbling means the amount and distribution of intramuscular fat in a cross section of musculus longissimus muscle [1]. In particular, eating quality traits such as taste, juiciness and tenderness of meat are influenced by the amount of intramuscular fat [2]. High levels of marbling improve the palatability and acceptability of beef by affecting the taste and tenderness of the meat [3, 4]. Therefore, the challenge to the beef cattle industry in Korea is the production of meat with higher marbling score. Knowledge on the genetic background of fat tissue accumulation and better understanding of the molecular mechanism of marbling are very important in beef production. To identify an informative gene or DNA marker for meat quality traits, candidate genes can be selected on the basis of the function of the encoded protein in physiological processes controlling energy homeostasis.

Recently, the *fat mass and obesity associated* (FTO) gene have been shown to have a relatively large effect on body mass index (BMI) and obesity-related traits in various human populations, suggesting that FTO associated with the development of fat tissue and adiposity [5–9]. Some studies also suggested that the FTO may play a key role in the regulation of energy homeostasis and associated with increased lipolytic activity in adipose tissue [9, 10]. In livestock species, polymorphic variations in the FTO gene are associated with fatness-related traits such as intramuscular fat deposition and backfat thickness [11–15]. In addition, the bovine FTO gene is located near the QTL region affecting meat quality traits on BTA18. Therefore, the FTO gene is considered as a positional and functional candidate gene for meat quality in beef cattle. However, most studies have so far been published for the pig. Moreover, the association analysis between single nucleotide polymorphisms (SNPs) within exons of the FTO gene and meat quality traits has not been reported in Hanwoo (Korean cattle). The objectives of our study were to identify new SNPs in the exonic regions of FTO gene and to evaluate the association of these SNPs with meat quality traits in Hanwoo.

## Methods

### Animals and genomic DNA extraction

A total of 300 steers, which were registered in a national database of cattle and guided with standardized breeding programs provided by the *Hoengseong* Hanwoo population in Korea, were used to genotype and collect carcass traits. To confirm genetic inheritance of the identified SNPs, 51 Hanwoo animals, which were used for the evaluation of performance ability for proven sires in the national progeny testing programs, were genotyped. The carcass data analyzed in the current study included marbling score (MS), meat color (MC), fat color (FC), meat texture (MT), meat maturity (MA), backfat thickness (BF), *Longissimus dorsi* muscle area (LMA), and carcass weight (CW). The means carcass traits values were 5.98 ± 1.73 for MS, 4.91 ± 0.37 for MC, 3.00 ± 0.10 for FC, 1.09 ± 0.28 for MT, 2.03 ± 0.19 for MA, 11.99 ± 4.01 mm for BF, 90.05 ± 9.57 cm^2^ for LMA and 427.11 ± 46.96 kg for CW.

Meat samples were collected from 13th thoracic rib to the first lumbar vertebrae of the steers within 24 hr of slaughter and evaluated according to the Animal Product Grading System of Korea. Genomic DNA was extracted from tail root-hair by using a NaCl precipitation protocol [16] with a slight modification. The DNA sample was suspended in TE buffer (10 mM Tris–HCl, pH 7.4; 1 mM EDTA) and stored at -20°C until analysis.

### Resequencing and SNP discovery

**Figure 1:**
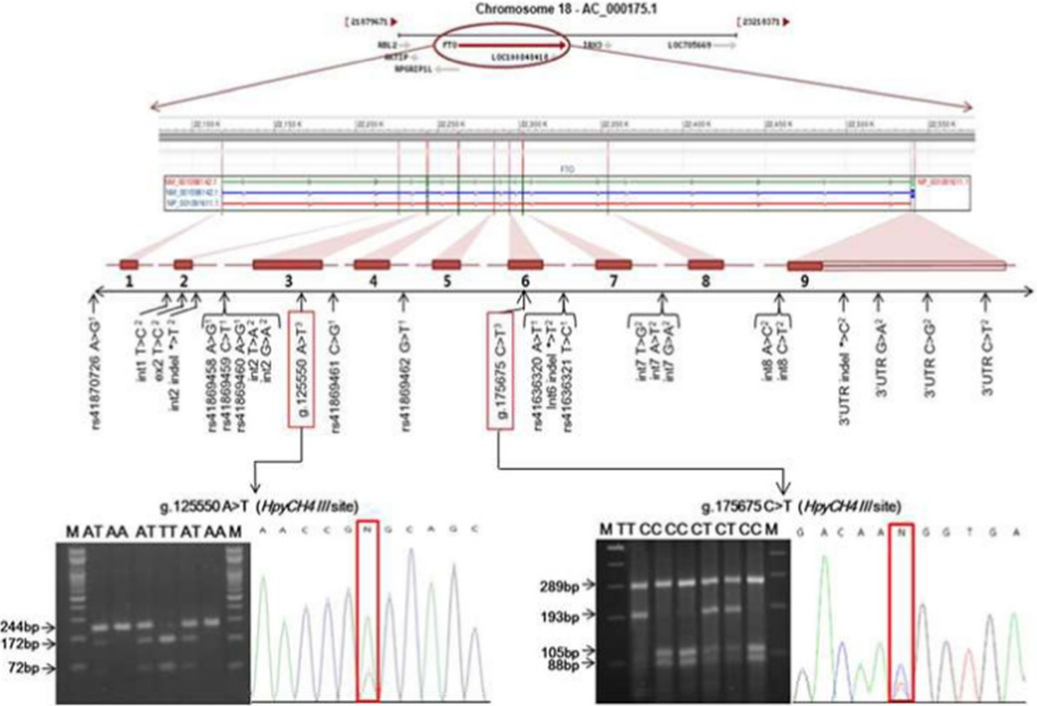
**New exonic SNPs identified within the FTO gene in the Hanwoo (Korean cattle);** ^**1**^ **Reported SNPs by NCBI dbSNP,** ^**2**^ **Reported SNPs by Horvat et al. [**[17]**],** ^**3**^ **We identified new SNPs within exonic regions of FTO gene in Hanwoo population.** New SNPs were genotyped using the PCR-RFLP method. For the RFLP analysis, amplified fragments were digested with restriction enzyme *HpyCH4* for g.125550A > T and g.175675C > T, respectively.

The bovine FTO gene is mapped to chromosome 18, which includes nine exons coding 505 amino acids (Figure 1). To identify polymorphisms within the coding region of the Hanwoo FTO gene, the nine pairs of primers (Table 1) were designed to amplify the all exon regions based on the genomic sequence of the bovine FTO gene from NCBI GenBank reference sequences (Genomic sequence: AC_000175.1 chromosome 18 reference *Bos taurus* UMD 3.1 primary assembly) using a web-based software Primer 3.0 program (http://frodo.wi.mit.edu/primer3). To determine SNP identification, pooled DNA samples from the sixty unrelated animals were amplified by PCR using the each primer pair. The PCR amplification was performed in a DNA thermal cycler (Perkin Elmer Cetus, Norwalk, CT). The PCR reaction was performed in a 20 μL reaction mixture containing 0.1 μM of each primer, 1.5 mM MgCl_2,_ 250 μM of each dNTP and 1 unit of *Taq* DNA polymerase, 10 X reaction buffer and 50 ng of pooled DNA as template. The PCR conditions were at 94°C for 5 min, followed by 35 cycles of 94°C for 30 s, annealing at 55°C to 57°C for each primer for 20 s and 72°C for 1 min, with a final extension at 72°C for 5 min. The PCR products were verified by 2% agarose gel electrophoresis and purified with Wizard Prep PCR purification kit (SolGent, Korea). The purified PCR amplicons were directly sequenced in both directions using BigDye™ Terminator V3.1 Cycle Sequencing Kit in an ABI PRISM 3730 Genetic Analyzer (Applied Biosystems, Foster City, CA, USA) according to the manufacturer's instructions. GENESCAN 3.7 ANALYSIS software (Applied Biosystems, USA) was used to assemble the sequences and to identify polymorphisms.

**Table 1:** **The primer sequence used to amplify the exonic sequence variants in the bovine FTO gene**

Primer	Primer sequences (5′-3′)	Amplified region	Fragment size (bp)	Location	GenBank accession no.
*FTO*-1	F-GGCTATAACGGCAGCATGAA	exon 1	844	4 ~ 847	*AC_000175.1*
	R-CCAGGTGGTGAAGATGGAAA				
*FTO*-2	F-AAGGCATTGTCAGCCTGGATG	exon 2	714	107821 ~ 108536	
	R-ATATGGTACAAGGAGCACCAGG				
*FTO*-3	F-TGTCTGTAATGGAGCTTGGACC	exon 3	906	124883 ~ 125788	
	R-CAATCATATCTCCAGAGCCTGC				
*FTO*-4	F-CCTTTGCAACTCTCTAGTCCTGTG	exon 4	1064	144220 ~ 145283	
	R-CTCTCCAAACTCCCCAAACTTC				
*FTO*-5	F-TTTCCAGGCAAGAGTACTGGAG	exon 5	675	166054 ~ 166728	
	R-CTTGACCTTGACCTTGACCTTG				
*FTO*-6	F-AACGTGGGGTTCACTTACTGTG	exon 6	651	175553 ~ 176203	
	R-GCAAGAATACTGGAGTGGGTTG				
*FTO*-7	F-CTGTCATGTGCTTTAGGATCTC	exon 7	617	183602 ~ 184218	
	R-TGTCTGTGAGTCAACTTCTGTT				
*FTO*-8	F-TAAGGCAGTGGAGCTTTGGA	exon 8	821	235720 ~ 236540	
	R-TGTGCACAGAAATGAGGCTG				
*FTO*-9	F-TCACAGTGGATTGCCAAGGT	exon 9	824	420877 ~ 421700	
	R-TGTGGCTCATGCAACTGAAG				

### SNP genotyping using PCR-RFLP

Two SNPs, g.125550A > T and g.175675C > T, newly identified in the exons 3 and 6 of the Hanwoo FTO gene respectively, were genotyped using PCR-RFLP method. PCR primers used for PCR-RFLP analysis were 5'-TTCCTCAAGCTCAACAGCTACC-3' and 5'-ACGGTTCCTCTTTCAGGTATGG-3' for the g.125550A > T SNP, and 5'-AGAGTCAGTTCTAGGTGCTGTGGT-3' and 5'-CCACAGTTCTCAGAAGCCCTTA-3' for the g.175675C > T SNP. PCR amplifications were carried out as for the SNP detection protocol. For the RFLP analysis, amplified fragments were digested with restriction enzyme *HpyCH4III* for g.125550A > T and g.175675C > T at 37°C for 3 h, respectively. The digested DNA fragments were separated on 2% agarose gel by electrophoresis with 1 X TBE buffer. The gels were stained with ethidium bromide (EtBr) and the fragments were visualized using a UV transilluminator (Ultra Rum Inc, USA). To define each genotype according to band patterns, the PCR products of different RFLP type corresponding to each genotype were sequenced and analyzed for nucleotide changes.

### Statistical analyses

Allele and genotype frequencies of SNPs and Hardy-Weinberg equilibrium were estimated and tested using PROC ALLELE (SAS Inst. Inc., Cary, NC, USA). Associations between SNP genotypes and phenotypes of carcass traits were analyzed with the following liner mixed model using the MIXED procedure of SAS (SAS Inst. Inc., Cary, NC, USA). The data were analyzed according to the following model: Y_*ijk*_ = *μ* + G_i_ + S_j_ + P_k_ + βA_l_ + e_ijkl, W_here Y_*ijk =*_the value of carcass traits; *μ* = the overall mean for each trait; G = fixed effect of single SNP marker genotype; S = random effect of sire, P = fixed effect of parity; A = fixed effect of age as a covariate; and e_ijkl_ = random residual effect. The *Bonferroni* correction for multiple testing was performed to remove any false positives. The additive and dominance genetic effects were also estimated using REG procedure of SAS according to Falconer and Mackay [18]. This research was followed by internationally recongnized guidelines (Institutional Animal Care and Use Committee, IACUC) for animal experiment.

## Results

In this study, we sequenced all exon regions of the bovine FTO gene and newly identified two exonic SNPs in Hanwoo steers (Figure 1). An A to T transition (g.125550A > T SNP) was located in exon 3 and a C to T substitution (g.175675C > T SNP) was located in exon 6 (GenBank accession no. AC_000175). Genotyping of the two novel SNPs in the coding region of the bovine FTO gene was performed by a PCR-RFLP method in Hanwoo population. The allele and genotype frequencies in the two novel SNPs of the FTO gene are shown in Table 2. The genotypic frequencies were as follows: 47.0% AA, 44.3% AT and 8.7% TT for the g.125550A > T SNP; 52.0% CC, 37.3% CT and 10.7% TT for the g.175675C > T SNP. The observed genotype distributions were in good agreement with those expected according to the Hardy-Weinberg equilibrium in this population. Overall average of heterozygosity (He) and polymorphic information contents (PIC) for two SNP markers were calculated to 0.576 and 0.486, respectively. The results of the association analysis for the FTO gene SNP markers with various carcass traits are presented in Table 3. The g.125550A > T SNP genotype was significantly associated with effects on MS. Animals with the AA or TT homozygous genotype had a significantly higher MS (*p* < 0.001) than the animals with AT heterozygous genotype and this was significant after *Bonferroni* correction of the significance threshold (*p* = 0.003). Dominance effect was also observed for the marbling score (P < 0.05) with higher marbling score of homozygous animals. However, no significant association was detected between the g.175675C > T SNP genotype and carcass traits measured in this study.

**Table 2:** **The genotypes and allele frequencies for SNP markers of the FTO gene in Hanwoo**

SNP marker	Frequency (%)					He	PIC	HWE	
	Genotype (No. of head)			Allele				χ ^2^	p-value
g.125550A > T	AA (141)	AT (133)	TT (26)	C	G	0.573	0.478	0.758	0.684
	47.0	44.3	8.7	69.3	30.7				
g.175675C > T	CC (156)	CT (112)	TT (32)	C	T	0.579	0.494	2.955	0.228
	52	37.3	10.7	70.7	29.3				

He: Heterozygosity, PIC: Polymorphic Information Content, HWE: Hardy-Weinberg Equilibrium.

**Table 3:** **The least square means and standard errors for carcass traits with genetic effects according to the FTO genotypes in Hanwoo population**

SNP	Traits ^1^	Genotype (mean ± SE)			P-value		Genetic effects	
					*P*_*raw*_	*P*_*corrected*_	Additive	Dominance
g.125550A>T		**AA**	**AT**	**TT**				
	MS/1 ~ 7	6.082 ± 0.194^a^	5.173 ± 0.200^b^	6.000 ± 0.461^a^	**<0.001**	**0.003**	0.082 ± 0.501	1.734 ± 0.641^***^
	MC/4 ~ 6	4.931 ± 0.033	4.956 ± 0.034	4.923 ± 0.079	0.846	1.246	0.008 ± 0.086	-0.058 ± 0.110
	FC/2 ~ 4	3.000 ± 0.009	2.958 ± 0.009	3.000 ± 0.022	0.539	0.922	-0.000 ± 0.024	0.028 ± 0.031
	MT/1 ~ 2	1.068 ± 0.036	1.159 ± 0.037	1.076 ± 0.086	0.209	0.415	-0.008 ± 0.094	-0.173 ± 0.120
	MA/2 ~ 3	2.054 ± 0.034	2.130 ± 0.035	2.153 ± 0.082	0.244	0.478	-0.099 ± 0.089	-0.052 ± 0.0114
	BF/mm	14.301 ± 0.614	14.797 ± 0.631	13.692 ± 1.455	0.730	1.013	0.609 ± 1.579	-1.600 ± 2.022
	LMA/cm^2^	92.671 ± 1.132	89.739 ± 1.165	91.230 ± 2.684	0.199	0.348	1.440 ± 2.913	4.423 ± 3.731
	CW/kg	431.045 ± 6.360	432.972 ± 6.541	430.923 ± 15.071	0.976	1.462	0.131 ± 16.358	-3.964 ± 20.947
g.175675C > T		**CC**	**CT**	**TT**				
	MS/1 ~ 7	5.932 ± 0.164	5.854 ± 0.258	5.571 ± 0.390	0.695	0.695	0.360 ± 0.424	-0.204 ± 0.668
	MC/4 ~ 6	2.991 ± 0.006	3.000 ± 0.010	3.000 ± 0.016	0.748	0.748	-0.008 ± 0.017	-0.008 ± 0.027
	FC/2 ~ 4	1.101 ± 0.027	1.062 ± 0.043	1.190 ± 0.065	0.272	0.272	-0.088 ± 0.071	0.167 ± 0.112
	MT/1 ~ 2	2.135 ± 0.027	2.062 ± 0.043	2.000 ± 0.065	0.097	0.097	0.135 ± 0.071	0.010 ± 0.112
	MA/2 ~ 3	30.694 ± 0.086	3.666 ± 0.135	3.476 ± 0.205	0.618	0.618	0.218 ± 0.222	-0.162 ± 0.351
	BF/mm	13.652 ± 0.442	14.125 ± 0.694	14.248 ± 1.049	0.782	0.782	-0.585 ± 1.139	-0.359 ± 1.796
	LMA/cm^2^	91.872 ± 0.874	91.666 ± 1.371	93.190 ± 2.074	0.815	0.815	-1.317 ± 2.251	1.730 ± 3.548
	CW/kg	431.525 ± 4.755	441.416 ± 7.456	431.428 ± 11.272	0.520	0.520	0.096 ± 12.235	-19.875 ± 19.289

^1^ MS, marbling score; MC, meat color; FC, fat color; MT, meat texture; MA, meat maturity; BF, backfat thickness; LMA, M. *Longissimus dorsi* muscle area; CW, carcass weight.

^***^P <0.001.

^a,b^Within a row, means with different superscripted letters are different (*P* <0.01).

## Discussion

The development of functional genomics has revealed a large number of genes related with fat accumulation and lipid metabolism. Several QTLs for carcass traits and a large number of potential candidate genes based on a known relationship with physiological or biochemical processes and carcass traits have been reported in beef cattle [19–21]. However, a limited number of genetic markers have been recognized for carcass and meat quality traits in beef cattle and these markers explain a relatively small proportion of the genetic variation for a limited number of traits [22]. Fatness related traits such as marbling are classified as a quantitative trait with a high contribution of genetic variation [23]. Thus, identification of DNA polymorphism associated with (or responsible for) fatness traits may be useful for marker assisted selection.

Many regulatory factor genes are involved in the formation of marbling [24], and increased knowledge about the relationship between these genes and the fat accumulation and lipid metabolism is of utmost importance for the improvement of meat quality in beef cattle. The FTO gene is a new candidate gene related to the development of fat tissue and obesity. This gene encodes 2-oxoglutarate-dependent oxygenases, which are involved in various processes, including DNA repair, fatty acid metabolism and posttranslational modifications and is highly expressed in the hypothalamic pituitary adrenal axis, which plays a key role in the control of energy balance [25]. Therefore, the polymorphic variation in this gene may play a causal role in the regulation of energy homeostasis or in the development of fat tissue [5, 26]. In addition, the FTO is a transcriptional co-activator, which facilitates transcription from unmethylated and methylation-inhibited gene promoters and enhances C/EBPs binding to DNA, and that it may play a role in the regulation of adiposity [15, 27]. These findings suggest close link between FTO and fat deposition and lipid metabolism. In human, genetic variation of this gene was extensively studied and had confirmed that a strong and highly significant association with fatness-related traits such as fat mass, obesity and body mass index in different populations [5–7]. This gene has also become one of the candidate genes for detecting polymorphism associated with the fat deposition in livestock species. Several studies on pig have shown the association between the polymorphism of the FTO gene and intramuscular fat (IMF) [11, 13, 28]. A significant association between g.276 T > G polymorphism of the 3'-untranslated region and intramuscular fat deposition was reported by Fontanesi et al. [28] in the Italian White Duroc breed. Fan et al. [12] also suggested significant association of the g.-1191A > G SNP in 5′ regulatory region of the porcine FTO gene and intramuscular fat content. These results provided that the porcine FTO gene might play an important function in intramuscular fat and not directly in subcutaneous or abdominal fat deposition [13]. Recently, some previous studies have reported the bovine FTO gene associated with carcass traits in beef cattle breeds. Wei et al. [14] have reported that g.1071C > T SNP in exon 5 within the bovine FTO gene was associated with backfat thinckness and *longissmus* muscle area traits in five Chinese native cattle breeds. In this study, however, the effect of genotypes of the two novel SNPs was not statistically significant for backfat thickness and *longissmus* muscle area. This might be supported by the fact that Hanwoo breed exhibits very low or negative genetic correlation between marbling and backfat thickness [29, 30]. Also, Jevšinek Skok et al. [31] have reported that the T > C SNP in exon 2 of the FTO gene showed a significant effect on growth and carcass traits such as live weight at slaughter, carcass weight and lean weight in Slovenian Simmental population. However, the associations between the SNPs of the FTO gene and intramuscular fat have not been reported in beef cattle. To the knowledge of the authors, the present study is the first report on association between exonic SNP marker within coding regions of the FTO gene and fatness-related traits such as marbling score in beef cattle, suggesting a possible effect of the gene on intramuscular fat deposition. The two exonic SNPs identified in this study by resequencing have not been reported previously and are therefore novel. The bovine FTO gene was near to the QTL region for carcass traits [32, 33]. As the A to C substitution in exonic SNP g.125550A > T does not result in an amino acid change due to synonymous mutation, this is unlikely to be the causal mutation for marbling and instead is likely to be genetically linked to nearby QTL or causative mutations that affect marbling score in beef cattle. The synonymous SNPs could affect gene function because they are under evolutionary pressure, alter the structure, function and expression level of proteins, and can affect mRNA splicing, stability and protein structure as well as folding [34].

## Conclusion

In conclusion, we identified two novel exonic SNPs of the FTO gene in Hanwoo population and the g.125550A > T SNP genotype showed significant effect on marbling score. These findings suggest that the FTO gene-specific SNP identified in this study may be useful molecular marker for selection to increase the levels of marbling in Hanwoo. This study also will contribute to a better understanding of the molecular mechanisms of marbling in beef cattle. Further studies are will be needed to confirm the associated effect on other population and breeds.
